# Dysentery

**Published:** 1852-05

**Authors:** 


ART. VII.—*Dysentery.*

*Messrs. Editors*:—The dysentery prevailed to a large extent in this region last autumn, attacking indiscriminately, from infancy to old age. I tried the various means recommended in books and journals, and used the same that was effectual in sporadic cases; gave opium and its preparations so as to produce its constitutional effects, and kept them under its influence for forty-eight hours, or more, without any good resulting. I then adopted a mixed plan of treatment, varied according to the condition of the patient, but the "general plan" was something like this: First, two or three powders, at intervals of three hours, composed of cal. and Dov. (to suit the case,) followed, by seidlitz powder, as a cathartic, and, after a sufficient evacuation of the bowels, gave a full dose of Doveri, and decoctum Hæmatoxyli, or the solution of the extract, *ad libitum*. I frequently used hot fomentations to the bowels and spts. Terebinthinæ. Under this treatment universal success followed speedily. I did not venesect, cup, leech, or blister, while pursuing the above plan. I think you will readily perceive that the means I used are those which are well suited to the pathological conditions present, and are in accordance with sound therapeutical reasoning.

Yours truly, Waters.

April 26th, 1852.

